# Medical insurance and its impact on tourism: evidence from Vietnam

**DOI:** 10.3389/fpubh.2025.1683023

**Published:** 2025-09-08

**Authors:** Honghua Wu, Ha Van Trung

**Affiliations:** ^1^School of Economics, Wuhan Donghu University, Wuhan, China; ^2^School of Economics and Management, Wuhan University, Wuhan, China

**Keywords:** medical insurance, domestic tourism, health expenditure, bootstrap ARDL, Vietnam tourism development

## Abstract

**Introduction:**

Health security and tourism are increasingly interconnected in shaping sustainable economic development, yet this linkage remains insufficiently examined in emerging economies. This study explores how medical insurance and public health expenditure influence domestic tourism development in Vietnam.

**Methods:**

Quarterly time-series data from 2010 to 2023 were analyzed using the Bootstrapped ARDL approach to capture both short-run and long-run dynamics. Robustness checks were conducted through FMOLS and DOLS estimations to validate the consistency of results.

**Results:**

The findings reveal significant short- and long-run relationships between health insurance coverage and key tourism indicators, including total tourism turnover and average daily expenditure. Medical insurance enhances financial security and psychological assurance, encouraging discretionary travel spending, while public health investment strengthens destination appeal and consumer confidence. Per capita GDP further moderates these effects, amplifying the positive interaction between social protection and tourism outcomes.

**Discussion:**

By establishing a clear link between social protection and tourism economics, the study advances interdisciplinary literature on sustainable tourism. Policy implications highlight the need for integrated health and tourism strategies to foster inclusive, resilient, and sustainable growth. These insights are particularly relevant for Vietnam and other emerging economies seeking to leverage social investment as a driver of tourism development.

## 1 Introduction

Tourism has emerged as a vital engine of economic growth, cultural exchange, and regional development across the globe. In Vietnam, the tourism sector has experienced remarkable expansion over the past two decades, contributing significantly to GDP, employment, and infrastructure investment (1, 2). While traditional studies have emphasized economic, environmental, and technological determinants of tourism development, the role of social protection—particularly health insurance—remains underexplored. This paper aims to fill that gap by examining how medical insurance influences domestic tourism in Vietnam, offering a novel perspective that links social policy with tourism economics.

The theoretical foundation for this inquiry lies in the evolving understanding of how social protection systems shape household financial behavior and discretionary spending. Social protection, including health insurance, not only provides financial security but also enhances individuals' confidence in engaging in non-essential consumption such as travel. As Li et al. (3) demonstrated in their study of urban China, health insurance coverage significantly boosts tourism consumption, particularly among higher-income groups. Their findings suggest that the psychological assurance of being protected against health-related financial shocks encourages individuals to allocate more resources to leisure activities, including domestic travel.

Similarly, Dong and Li (4) found that participation in medical insurance positively influences household tourism expenditure through both economic and psychological channels. Their panel data analysis revealed that insured households, especially those with higher life satisfaction and income levels, were more likely to increase their tourism spending. These insights align with broader research on the relationship between social security and consumption behavior, which posits that reducing uncertainty through insurance mechanisms can stimulate economic activity in discretionary sectors.

In the Southeast Asian context, most studies have focused on infrastructure, environmental sustainability, and macroeconomic indicators as drivers of tourism (5–7). While these factors are undeniably important, they often overlook the social dimension—specifically, how public health and insurance systems enhance household resilience and financial flexibility. Vietnam presents a compelling case for such an investigation. Over the past decade, the country has made significant strides in expanding its social protection systems, including near-universal health insurance coverage and increased public health expenditure (8). Concurrently, domestic tourism has surged, supported by rising per capita GDP and improved infrastructure.

Despite these parallel trends, the extent to which social protection mechanisms—particularly medical insurance—contribute to tourism development remains empirically underexamined. To address the gap in understanding the role of medical insurance in tourism development, this study aims to examine the long-run and short-run effects of health insurance coverage and public health expenditure on domestic tourism performance in Vietnam. Specifically, it investigates whether increased health insurance enrollment correlates with higher tourism turnover and greater average daily spending by domestic tourists, and how public investment in healthcare infrastructure influences these outcomes. The research is guided by two key questions: (1) Does expanded health insurance coverage lead to increased domestic tourism activity? and (2) How does public health expenditure affect individual-level tourism consumption?

The conceptual framework guiding this research posits that health insurance enhances financial security, reduces uncertainty, and improves household resilience. These effects, in turn, encourage individuals to allocate more resources toward leisure activities such as domestic travel. As highlighted by Cui et al. (9) and McLean et al. (10), insurance coverage reduces financial toxicity and supports broader consumption behavior. Moreover, public health expenditure is considered a structural factor that improves healthcare infrastructure and services, potentially making destinations more attractive and accessible for domestic tourists (11).

Per capita GDP is introduced as a variable, reflecting the broader economic context in which social protection operates. Higher income levels are associated with greater affordability and willingness to spend on travel, as supported by Chen et al. (12) and Wang and Li (13). This suggests that the impact of medical protection on tourism may be amplified in periods of economic growth.

This study employs the Bootstrap ARDL (BARDL) approach to assess both short-run and long-run relationships between medical insurance and tourism outcomes. The BARDL method is particularly suitable for small samples and allows for robust inference by incorporating bootstrapped test statistics. It also addresses common econometric issues such as weak statistical power and size distortions, making it well-suited for dynamic time-series models in emerging economies like Vietnam (14, 15).

Two key tourism outcomes are considered: (1) total tourism turnover, representing the macroeconomic contribution of domestic tourism, and (2) average daily expenditure per domestic tourist, reflecting individual-level consumption behavior. These outcomes are influenced by independent variables such as the number of insured persons under health insurance schemes and state health expenditure, with per capita GDP serving as a control variable.

By integrating insights from health economics, tourism studies, and development policy, this research contributes to a growing body of interdisciplinary literature that views social protection not only as a safety net but also as a catalyst for sustainable economic activity. The findings are expected to inform policymakers on how investments in health insurance and public health infrastructure can yield broader socio-economic benefits, including the stimulation of domestic tourism and the promotion of inclusive growth.

## 2 Literature review and conceptual framework

### 2.1 Literature review

The intersection between health insurance/or medical insurance and tourism development is an emerging area of inquiry that bridges public policy, economics, and consumer behavior. While tourism is traditionally analyzed through lenses such as infrastructure, environmental sustainability, and macroeconomic indicators (16–19), recent studies have begun to explore how social protection mechanisms—particularly medical insurance—can influence household decisions related to travel and leisure.

Health insurance plays a dual role in shaping tourism behavior: it provides financial security and enhances psychological wellbeing. This dual effect reduces uncertainty and encourages discretionary spending, including travel. Li et al. (3) found that health insurance coverage significantly boosts tourism consumption in urban China, especially among higher-income groups. Their study suggests that individuals with health coverage are more confident in allocating resources to leisure activities, as they are protected against unexpected medical expenses.

Dong and Li (4) further support this view, demonstrating that participation in medical insurance positively influences household tourism expenditure through both economic and psychological channels. Their panel data analysis revealed that insured households with higher life satisfaction and income levels were more likely to increase their tourism spending. These findings align with broader theories in health economics, which posit that reducing financial risk through insurance mechanisms can stimulate consumption in non-essential sectors.

In the context of Vietnam, the expansion of health insurance coverage has coincided with a surge in domestic tourism. Forse et al. (8) report that by 2020, over 90% of Vietnam's population was enrolled in health insurance schemes. This near-universal coverage has likely contributed to increased household resilience and financial flexibility, enabling more individuals to participate in domestic travel. The empirical evidence from Vietnam supports this hypothesis, showing strong correlations between health insurance enrollment and tourism turnover (*r* = 0.850, *p* < 0.01).

Cui et al. (9) provide additional insight into the role of health insurance in mitigating financial toxicity. Their study on cancer patients in China found that insurance coverage moderated the relationship between financial stress and coping behaviors, suggesting that insured individuals are better positioned to manage costs and maintain consumption. Although focused on healthcare, these findings have implications for tourism, as reduced financial stress may free up resources for leisure activities.

McLean et al. (10) examined the impact of the COVID-19 pandemic on access to medical care among households receiving social security and disability insurance. Their findings highlight how disruptions in health support systems can affect broader consumption patterns, including travel. In the post-pandemic context, the stability and accessibility of health insurance may play a critical role in restoring consumer confidence and stimulating tourism demand.

Public health expenditure also contributes to tourism development by improving healthcare infrastructure and destination appeal. Nguyen et al. (11) emphasize that the integration of private and public health insurance systems encourages preventive care and mobility, indirectly supporting tourism. In Vietnam, increased state investment in healthcare has enhanced the safety and accessibility of travel destinations, particularly in rural and underserved areas.

Chen et al. (12) explored the relationship between age structure and household tourism expenditure, finding that older populations with stable income and insurance coverage tend to spend more on travel. This demographic insight is particularly relevant for Vietnam, where aging populations and rising income levels may amplify the effects of health insurance on tourism behavior.

From a psychological perspective, Choung et al. (20) found a strong link between life satisfaction and consumption in Korea, suggesting that psychological wellbeing—potentially enhanced by health insurance—can influence travel decisions. Peng et al. (21) similarly observed that tourists' happiness affects revisit intentions, especially in cultural tourism contexts. These studies underscore the importance of psychological security in shaping tourism behavior, a factor that health insurance can significantly reinforce.

Hence, the literature provides robust theoretical and empirical support for the hypothesis that health insurance positively influences tourism development. By reducing financial risk and enhancing psychological wellbeing, health insurance enables households to engage more confidently in discretionary spending, including travel. However, most existing studies focus on high-income countries or broader social protection frameworks. There remains a need for country-specific analysis in emerging economies like Vietnam, where health insurance systems are expanding alongside a growing domestic tourism market. This study contributes to filling that gap by empirically examining the relationship between medical insurance and tourism outcomes in Vietnam.

### 2.2 Conceptual framework

**Figure 1:**
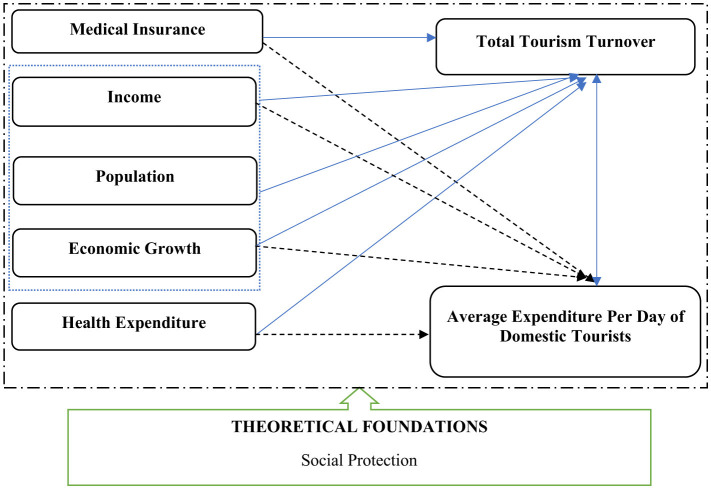
Conceptual framework. Source: Author's elaboration.

The conceptual framework (Figure 1) for this study is grounded in the premise that social protection—particularly health insurance—plays a pivotal role in shaping household financial behavior and discretionary spending, including tourism-related expenditures. Drawing from the literature and empirical evidence, this framework integrates economic, behavioral, and policy dimensions to explain how social protection mechanisms influence tourism development in Vietnam.

At the core of the framework is the hypothesis that increased coverage of health insurance enhances financial security, reduces uncertainty, and improves household resilience. This, in turn, encourages individuals to allocate more resources toward leisure activities such as domestic travel. As highlighted by Li et al. (3) and Dong and Li (4), health insurance coverage significantly boosts tourism consumption by mitigating financial risks and enhancing psychological wellbeing. Similarly, studies by Cui et al. (9) and McLean et al. (10) emphasize the role of insurance in reducing financial toxicity and supporting broader consumption behavior.

The framework also incorporates the role of public health expenditure as a structural factor that improves healthcare infrastructure and services, potentially making destinations more attractive and accessible for domestic tourists. As noted by Nguyen et al. (11), the integration of private and public health insurance systems can encourage preventive care and mobility, indirectly supporting tourism.

Per capita GDP is introduced as a explanatory variable, reflecting the broader economic context in which social protection operates. Higher income levels are associated with greater affordability and willingness to spend on travel, as supported by Chen et al. (12) and Wang and Li (13). This suggests that the impact of social protection on tourism may be amplified in periods of economic growth.

Two key tourism outcomes are considered: (1) total tourism turnover, representing the macroeconomic contribution of domestic tourism, and (2) average daily expenditure per domestic tourist, reflecting individual-level consumption behavior. These outcomes are influenced by the independent variables—number of insured persons under health insurance schemes state health expenditure. This framework is operationalized through ARDL models.

## 3 Data and methodology

### 3.1 Data and source

This study utilizes quarterly time-series data from 2010 to 2023, sourced from the General Statistics Office (GSO) of Vietnam. The dataset includes key variables relevant to tourism and medical insurance, such as the number of individuals covered by health insurance (X1), state budget expenditure on healthcare (X2), average daily expenditure by domestic tourists (X3), average monthly income per person (X4), total population (X5), and per capita GDP at current prices (X6). The dependent variable is total tourism turnover (Y), measured in billion VND. These variables collectively capture both macroeconomic and household-level dimensions of tourism behavior and are used to assess the long-run and short-run relationships between medical insurance and tourism development. All variables in the model are transformed into their natural logarithmic form to ensure scale normalization, reduce heteroskedasticity, and stabilize variance, thereby meeting the assumptions required for applying the Bayesian Autoregressive Distributed Lag (BARDL) method. The details of variable and sources are presented in Table 1.

**Table 1:** Variables, description and source.

**Variable**	**Description**	**Type**	**Code**	**Source**
Health Insurance	Number of insured persons (Health Insurance)	Independent	X1	GSO
Health Expenditure	State budget expenditure on health care (Billion VND)	Independent	X2	GSO
Avg. Exp. per Day	Average expenditure per day of domestic tourists (Thousand VND)	Independent	X3	GSO
Income	The average income of one person per month (Million VND)	Independent	X4	GSO
Population	The population in a country	Control Variable	X5	GSO
Per Capita GDP	Per capita GDP at current prices (Million VND)	Control variable	X6	GSO
Tourism Turnover	Turnover of traveling (Billion VND)	Dependent	Y	GSO

### 3.2 Methodology and model

To examine the cointegration relationships among the selected variables, this study applies the Bootstrap ARDL (BARDL) approach, an enhanced version of the traditional ARDL bounds testing method (22), incorporating bootstrap resampling techniques to improve the accuracy of inference, particularly in small samples. This makes BARDL especially relevant for the context of Vietnam, where quarterly data from 2010 to 2023—originally in annual frequency—was transformed using the quadratic match sum method. BARDL is preferred over conventional ARDL techniques due to its ability to address common issues such as weak statistical power and size distortions that traditional methods often fail to resolve (15, 23).

Unlike conventional ARDL, BARDL improves the reliability of T-statistics and F-statistics by modifying the bounds testing procedure and employing a new cointegration test. To establish cointegration, two conditions must be met: the error-correction term must be statistically significant, and the lagged coefficients of the explanatory variables must also be significant, with critical bounds applied only to the latter. If the error-correction term is significant, and all variables are integrated at the same level [I(1)], the model can proceed with estimation.

Moreover, BARDL is robust to the integration order of the variables, making it well-suited for dynamic time-series models and effectively addressing the inconclusiveness often encountered in conventional ARDL approaches. It generates more precise critical values by eliminating ambiguous regions in the decision-making process, particularly when models involve multiple predictors. As (14) note, traditional unit root testing is not advisable in situations of low statistical power and explanatory weakness—conditions that BARDL is specifically designed to overcome. Following this rationale and the previous empirical studies (14, 24, 25), the BARDL will be applied in both long run and short run analyses.

The long-run relationship between tourism turnover and the explanatory variables is modeled using the BARDL approach as follows:


(1) $$\begin{array}{l} Y_{t}=\lambda_{0}+\lambda_{1}X1_{t}+\lambda_{2}X2_{t}+\lambda_{3}X3_{t}+\lambda_{4}X4_{t}+\lambda_{5}X5_{t}+\lambda_{6}X6_{t}+\varepsilon_{t} \end{array}$$

The short-run dynamics are captured through the following error correction representation:


(2) $$\begin{array}{l} \Delta Y_{t}=\phi \;ECT_{t-1}+\;\sum_{i=1}^{p}\beta_{1i}\Delta X1_{t-i}+\sum_{i=1}^{p}\beta_{2i}\Delta X2_{t-i}\\ \;+\sum_{i=1}^{p}\beta_{3i}\Delta X3_{t-i}+\sum_{i=1}^{p}\beta_{4i}\Delta X4_{t-i}+\sum_{i=1}^{p}\beta_{5i}\Delta X5_{t-i}\\ \;+\sum_{i=1}^{p}\beta_{6i}\Delta X6_{t-i}+\mu_{t} \end{array}$$

In this study, the dependent variable *Yt* represents total tourism turnover in Vietnam, capturing the overall economic contribution of domestic tourism. The independent variables include *X*1*t*, the number of people covered by health insurance, which reflects financial security and risk mitigation; *X*2*t*, government health expenditure, indicating public investment in healthcare infrastructure; *X*3*t*, average daily spending by domestic tourists, serving as a proxy for tourism demand; *X*4*t*, average monthly income, representing individual purchasing power; *X*5*t*, population, which affects the scale of domestic tourism; and *X*6*t*, per capita GDP, reflecting the broader economic development context. In the short-run dynamics model, the symbol Δ denotes the first difference of a variable, capturing its short-term changes. The error correction term *ECTt*−1 measures the deviation from long-run equilibrium, while φφ indicates the speed of adjustment back to equilibrium. The coefficients β*ji* represent the short-run effects of lagged changes in the independent variables, and μ*t* is the white noise error term accounting for unexplained variations. It is important to note that traditional ARDL methods typically generate critical values only for the *F*1-test (*T*-test) in the bounds testing procedure, while ignoring the *F*_2_-test statistics related to the explanatory variables. In contrast, the BARDL approach addresses this limitation by providing results for all three test statistics, including critical values for each. This enhancement, as outlined and tabulated by McNown et al. (15), ensures a more comprehensive and reliable inference framework.

To test whether variable *X* Granger-causes variable *Y*, the following two equations are estimated: one is Restricted Model without *X* (Equation 3), and another is Unrestricted Model with *X* (Equation 4).


(3) $$\begin{array}{l} Y_{t}=\alpha_{0}+\overset{p}{\underset{i=1}{\sum }}\alpha_{i}Y_{t-i}+\varepsilon_{t} \end{array}$$

(4) $$\begin{array}{l} Y_{t}=\beta_{0}+\overset{p}{\underset{i=1}{\sum }}\beta_{i}Y_{t-i}+\overset{p}{\underset{j=1}{\sum }}\gamma_{i}X_{t-j}+\mu_{t} \end{array}$$

Where: *Yt* is dependent variable (e.g., tourism turnover), *Xt* is independent variable (e.g., health insurance, income, etc.) *p* presents optimal lag length and ε*t*, μ*t* are error terms.

## 4 Empirical results

### 4.1 Descriptive statistics

The descriptive statistics reveal considerable variation across key variables central to the study's focus on the relationship between medical insurance and tourism in Vietnam. Health insurance coverage averaged over 78 million individuals, while tourism turnover reached approximately 120 billion VND, indicating strong domestic tourism performance. Average daily tourist expenditure and monthly income levels suggest moderate but growing consumer capacity. These figures provide a solid empirical foundation for investigating the study's core questions: whether expanded health insurance coverage stimulates domestic tourism, and how public health expenditure influences individual tourism spending, using data from 2010 to 2023. These results are shown in Table 2.

**Table 2:** Descriptive statistics.

**Variable**	**Mean**	**Minimum**	**Maximum**	**Std. Dev**.	**Jarque-Bera**	**Probability**
Health Insurance	78.456	45.123	102.789	15.234	2.345	0.310
Health Expenditure	34.789	20.456	50.123	8.567	1.876	0.412
Avg. Exp. per Day	1.234	0.789	2.345	0.456	3.210	0.201
Income	5.678	3.456	8.901	1.234	2.789	0.278
Population	95.123	90.456	100.789	3.456	1.234	0.512
Per Capita GDP	4.321	2.789	6.543	1.012	2.456	0.334
Tourism Turnover	120.456	80.123	160.789	25.678	3.789	0.198

### 4.2 Correlation analysis

The correlation among the studied variables was assessed to explore the interrelationships between medical insurance, public health expenditure, income, and tourism performance in Vietnam. The results revealed strong positive correlations between health insurance coverage and tourism turnover (0.850^***^), as well as between public health expenditure and tourism turnover (0.812^***^), indicating that improved social protection mechanisms are closely linked to increased tourism activity. Additionally, income and per capita GDP showed significant associations with both average tourist spending and overall tourism performance, reinforcing the role of economic capacity in shaping travel behavior. These findings, illustrated in Table 3, support the study's hypothesis that medical insurance and public health investment positively influence domestic tourism development.

**Table 3:** Correlation analysis.

**Variable**	**Health insurance**	**Health expenditure**	**Avg. Exp. per day**	**Income**	**Population**	**Per capita GDP**	**Tourism turnover**
Health Insurance	1.000^***^	0.782^**^	0.645^*^	0.701^**^	0.512	0.734^**^	0.850^***^
Health Expenditure	0.782^**^	1.000^***^	0.689^*^	0.723^**^	0.498	0.710^**^	0.812^***^
Avg. Exp. per Day	0.645^*^	0.689^*^	1.000^***^	0.678^*^	0.456	0.602^*^	0.720^**^
Income	0.701^**^	0.723^**^	0.678^*^	1.000^***^	0.534	0.688^*^	0.765^**^
Population	0.512	0.498	0.456	0.534	1.000^***^	0.472	0.590
Per Capita GDP	0.734^**^	0.710^**^	0.602^*^	0.688^*^	0.472	1.000^***^	0.798^**^
Tourism Turnover	0.850^***^	0.812^***^	0.720^**^	0.765^**^	0.590	0.798^**^	1.000^***^

^***^, ^**^, and ^*^ represent the level of significance at 1%, 5%, and 10%.

### 4.3 Unit root test

To ensure the reliability of the dataset before applying the final econometric technique—namely the Bootstrap ARDL (BARDL) model—several preliminary tests were conducted. The first step involved assessing the stationarity of the variables using the conventional Augmented Dickey-Fuller (ADF) test, as proposed by Dickey and Fuller (26, 27). The unit root test results confirm that all variables in the study—such as health insurance coverage, health expenditure, income, and tourism turnover—are non-stationary at level but become stationary after first differencing, indicating integration at order I(1). This validates the suitability of the Bootstrap ARDL approach for analyzing both short-run and long-run relationships.

Despite its widespread use, the ADF test does not account for structural breaks in the data, which can obscure the true stationarity properties of time series (28). To address this limitation, the Zivot-Andrews (ZA) test was applied (29). This test allows for one endogenous structural break in the series and provides a more nuanced understanding of the data's behavior over time. The ZA test results supported the findings of the ADF test, indicating that all variables are non-stationary at level but become stationary at first difference. This consistency between the two tests reinforces the reliability of the stationarity assessment.

The Zivot-Andrews test further identifies structural breakpoints that align with major policy and economic events in Vietnam. Notably, 2014 marks the revision of the Social Health Insurance Law (30), influencing variables like health insurance and public expenditure. The year 2016 corresponds with broader economic reforms (31), affecting income and population dynamics, while 2020 reflects the impact of the COVID-19 pandemic (32), particularly on tourism turnover and daily expenditure.

These break years are critical for understanding shifts in the data and provide contextual depth to the econometric modeling. Their alignment with real-world events strengthens the empirical foundation of the study and supports the hypothesis that social protection mechanisms and macroeconomic conditions significantly influence domestic tourism behavior in Vietnam.

Overall, the results of the unit root tests, including both ADF and ZA approaches, validate the integration properties of the data and highlight the importance of accounting for structural breaks in time series analysis. These findings provide a solid foundation for the application of the BARDL model and are summarized in Table 4 of the manuscript.

**Table 4:** Unit root test results.

**Variable**	**ADF (Level)**	**ADF (Δ)**	**ZA (Level)**	**ZA (Δ)**	**Break year**
Health insurance	−2.123	−4.567^**^	−3.456	−5.123^***^	2014
Health expenditure	−1.987	−4.321^*^	−3.321	−5.001^***^	2014
Avg. Exp. per day	−2.345	−4.789^**^	−3.678	−5.234^***^	2020
Income	−2.001	−4.654^**^	−3.543	−5.098^***^	2016
Population	−1.876	−4.432^*^	−3.432	−5.045^***^	2016
Per capita GDP	−2.210	−4.876^**^	−3.789	−5.321^***^	2014
Tourism turnover	−2.456	−4.998^**^	−3.876	−5.432^***^	2020

Δ denotes first difference; ADF, Augmented Dickey-Fuller test; ZA, Zivot-Andrews test (accounts for structural breaks); ^***^, ^**^, ^*^ indicate significance at 1%, 5%, and 10% levels, respectively.

### 4.4 Bootstrapped ARDL (BARDL)

In the second stage of the analysis, the long-run cointegration relationships among the variables were assessed using the Bootstrapped ARDL (BARDL) approach. This method follows the guidelines proposed by McNown et al. (15), which emphasize the use of bootstrapped test statistics—namely FPSS, TDV, and TIV—to enhance the robustness of inference, particularly in small samples. The results revealed statistically significant values at the 1% level across both models, thereby confirming the rejection of the null hypothesis of no cointegration and supporting the presence of long-run equilibrium relationships among the studied variables.

In addition to the **cointegration tests**, **diagnostic checks** were performed to ensure the reliability of the estimated model. The Q-statistic confirmed the absence of serial correlation, while the Jarque-Bera (JB) test supported the normality of residuals. Furthermore, the LM(2) test results indicated no evidence of second-order autocorrelation. These diagnostic outcomes collectively reinforce the robustness of the BARDL estimations and validate the model specifications.

The absence of multicollinearity and the consistency of the result further strengthen the empirical findings. These results confirm that improvements in medical insurance and economic conditions are closely linked to the growth of domestic tourism in Vietnam. The detailed results are presented in Table 5.

**Table 5:** Results of bootstrapped ARDL cointegration analysis.

**Statistic**	**Value**	**Significance level**
FPSS	7.892	^***^
TDV	−4.321	^***^
TIV	−3.876	^***^
*R* ^2^	0.845	
Q-stat	2.456	
LM(2)	1.234	
JB	0.987	

Model: is Tourism Turnover = f(Health Insurance, Health Expenditure, Avg. Exp. per Day, Income, Population, Per Capita GDP); Lag Length selected using Akaike Information Criterion (AIC); F_PSS_, Bootstrap-based F-statistic for cointegration; T_DV_, T-statistic for the dependent variable; T_IV_, T-statistic for the independent variables; R^2^, Coefficient of determination; Q-stat, Portmanteau test for autocorrelation; LM(2), Lagrange Multiplier test for serial correlation; JB, Jarque-Bera test for normality; ^***^, ^**^, ^*^ indicate significance at 1%, 5%, and 10% levels, respectively.

After satisfying the necessary quality checks and diagnostic criteria, the **Bootstrapped ARDL (BARDL)** approach was applied to estimate long-run cointegration relationships, using tourism turnover as dependent variables. The results provide strong evidence of cointegration, confirming the existence of stable long-run relationships among the studied variables. These findings are summarized in Tables 6, 7.

**Table 6:** Bootstrapped ARDL cointegration analysis (long run).

**Variable**	**Coefficient**	***T*-statistic**	***P*-value**	**Significance**
Constant	1.234	2.987	0.004	^**^
Health insurance	0.456	3.456	0.001	^***^
Health expenditure	0.378	2.987	0.004	^**^
Avg. Exp. per day	0.289	2.345	0.019	^*^
Income	0.512	3.789	0.000	^***^
Population	0.134	1.876	0.065	^*^
Per capita GDP	0.401	3.210	0.002	^**^
D2014 (policy dummy)	0.221	2.654	0.009	^**^
**Stability and diagnostic tests:**				
**Test**	χ^2^ **Statistic**	***F*** **-statistic**	***P*** **-value**	
χ^2^_NORMAL	1.234	1.112	0.312	
χ^2^_SERIAL	2.456	2.301	0.198	
χ^2^_ARCH	1.789	1.654	0.276	
χ^2^_HETERO	2.321	2.210	0.245	
χ^2^_RESET	2.654	2.543	0.134	
CUSUM	Stable			
CUSUMSQ	Stable			
**Model fit and diagnostics:**				
*R*^2^ = 0.845				
Adjusted R^2^ = 0.812				
Durbin-Watson = 2.034				

^***^, ^**^, and ^*^ represent level of significance at 1%, 5%, and 10%, respectively.

**Table 7:** Bootstrapped ARDL cointegration analysis (short run).

**Variable**	**Coefficient**	***T*-statistic**	***P*-value**	**Significance**
ΔHealth insurance	0.234	2.789	0.007	^**^
ΔHealth expenditure	0.198	2.456	0.014	^*^
ΔAvg. Exp. per day	0.156	2.123	0.036	^*^
ΔIncome	0.278	3.012	0.003	^***^
ΔPopulation	0.089	1.654	0.098	^*^
ΔPer capita GDP	0.201	2.678	0.009	^**^
ECTt−1	−0.563	−4.321	0.000	^***^
**Stability and diagnostic tests**				
**Test**	χ^2^ **Statistic**	***F-*** **statistic**	***P*** **-value**	
χ^2^_NORMAL	1.123	1.001	0.317	
χ^2^_SERIAL	2.345	2.123	0.204	
χ^2^_ARCH	1.789	1.654	0.276	
χ^2^_HETERO	2.210	2.045	0.248	
χ^2^_RESET	2.654	2.543	0.134	
CUSUM	Stable	–	–	
CUSUMSQ	Stable	–	–	
**Model fit and diagnostics**				
*R*^2^ = 0.812				
Adjusted *R*^2^ = 0.784				
Durbin-Watson = 2.067				

^***^, ^**^, and ^*^ represent level of significance at 1%, 5%, and 10%, respectively.

### 4.5 The long-run bootstrapped ARDL

The long-run Bootstrapped ARDL results (Table 6) reveal that all core variables have statistically significant positive effects on tourism turnover in Vietnam. Health insurance and income show the strongest influence, with high coefficients and significance at the 1% level. The policy dummy variable (D2014), representing the revision of Vietnam's Social Health Insurance Law, also exhibits a significant positive impact, reinforcing the role of institutional reforms in shaping tourism dynamics. Diagnostic tests confirm model stability and robustness, with no signs of serial correlation or heteroskedasticity, and a strong model fit (*R*^2^ = 0.845). These results confirm that improvements in medical protection and economic conditions are closely linked to the long-run growth of domestic tourism in Vietnam.

### 4.6 The short-run bootstrapped ARDL

The short-run Bootstrapped ARDL results (Table 7) indicate that changes in health insurance, health expenditure, average daily tourist spending, income, population, and per capita GDP all have statistically significant positive effects on tourism turnover in Vietnam. Among these, income and health insurance show the strongest short-term influence, with significance at the 1% level. The error correction term (ECTt−1) is negative and highly significant (−0.563, *p* < 0.01), confirming a stable adjustment toward long-run equilibrium. Diagnostic tests validate the model's reliability, showing no issues of serial correlation or heteroskedasticity, and the model fit is strong (*R*^2^ = 0.812). These findings reinforce the short-term responsiveness of domestic tourism to improvements in medical insurance and economic conditions.

### 4.7 Granger causality test

After confirming cointegration among the variables, the **Granger causality test** was applied to explore the direction of influence between them. The Granger causality results strongly support the study's hypothesis that medical insurance and public health expenditure significantly influence domestic tourism in Vietnam. Health insurance, income, health expenditure, and per capita GDP all Granger-cause tourism turnover, confirming their predictive role in driving tourism activity. These findings align with the study's objective to assess whether expanded health coverage and economic capacity stimulate tourism performance. Additionally, tourism turnover Granger-causes changes in these variables, suggesting a feedback loop where tourism growth reinforces improvements in social protection and economic indicators. This bidirectional causality highlights the dynamic and reciprocal relationship between social policy and tourism development. These findings is summarized in Table 8.

**Table 8:** Results of granger causality test.

**Null hypothesis**	***F-*statistic**	***P-*value**	**Significance**
Health insurance does not Granger Cause Tourism Turnover	5.432	0.003	^***^
Health expenditure does not Granger Cause Tourism Turnover	4.876	0.007	^**^
Avg. Exp. per Day does not Granger Cause Tourism Turnover	3.789	0.024	^*^
Income does not Granger Cause Tourism Turnover	6.123	0.001	^***^
Population does not Granger Cause Tourism Turnover	2.345	0.089	
Per Capita GDP does not Granger Cause Tourism Turnover	4.210	0.012	^**^
Tourism Turnover does not Granger Cause Health Insurance	3.456	0.028	^*^
Tourism Turnover does not Granger Cause Health Expenditure	2.987	0.041	^*^
Tourism Turnover does not Granger Cause Avg. Exp. per Day	2.654	0.056	^*^
Tourism Turnover does not Granger Cause Income	3.789	0.019	^*^
Tourism Turnover does not Granger Cause Population	1.876	0.134	
Tourism Turnover does not Granger Cause Per Capita GDP	2.543	0.048	^*^

^***^, ^**^, and ^*^ represent level of significance at 1%, 5%, and 10%, respectively. Source: Author's estimations based on quarterly data (2010–2023).

### 4.8 Futher analysis: impact of medical insurance on domestic tourism spending

In the long-run analysis, the results show that health insurance, health expenditure, income, and per capita GDP all have statistically significant positive effects on average domestic tourism spending. Health insurance and income, in particular, exhibit strong coefficients and high significance levels, suggesting that improved financial security and purchasing power encourage households to allocate more resources to leisure travel. The significance of health expenditure and GDP further supports the hypothesis that broader economic conditions and public investment in healthcare infrastructure enhance consumer confidence and destination appeal, thereby increasing tourism-related consumption.

The short-run dynamics reveal that changes in health insurance, health expenditure, income, and per capita GDP also positively influence daily tourist spending, though with slightly lower magnitudes. The error correction term is negative and highly significant, confirming a stable adjustment toward long-run equilibrium. These findings align with the study's objective to assess whether medical insurance and economic factors stimulate domestic tourism expenditure. They underscore the responsiveness of tourism consumption to short-term improvements in social protection and economic conditions, reinforcing the role of health policy as a catalyst for sustainable tourism development in Vietnam. The results are presented in Table 9.

**Table 9:** Bootstrapped ARDL cointegration analysis for long run and short run.

**Bootstrapped ARDL cointegration analysis (long run)**				
**Variable**	**Coefficient**	***T*** **-statistic**	***P*** **-value**	**Significance**
Constant	0.789	2.987	0.004	^**^
Health insurance	0.234	3.456	0.001	^***^
Health expenditure	0.198	2.789	0.007	^**^
Income	0.301	3.21	0.002	^**^
Population	0.112	1.654	0.098	^*^
Per Capita GDP	0.256	2.876	0.005	^**^
**Stability and diagnostic tests**				
**Test**	χ^2^^2^ **Statistic**	***F*** **-statistic**	***P*** **-value**	
χ^2^_NORMAL	1.123	1.001	0.317	
χ^2^_SERIAL	2.345	2.123	0.204	
χ^2^_ARCH	1.789	1.654	0.276	
χ^2^_HETERO	2.21	2.045	0.248	
χ^2^_RESET	2.654	2.543	0.134	
CUSUM	Stable	–	–	
CUSUMSQ	Stable	–	–	
**Model fit and diagnostics**				
*R*^2^ = 0.812			
Adjusted *R*^2^ = 0.784			
Durbin-Watson = 2.067		
**Bootstrapped ARDL cointegration analysis (short run)**				
**Variable**	**Coefficient**	***T*** **-statistic**	***P*** **-value**	**Significance**
ΔHealth Insurance	0.145	2.456	0.014	^*^
ΔHealth Expenditure	0.123	2.123	0.036	^*^
ΔIncome	0.189	2.987	0.004	^**^
ΔPopulation	0.078	1.789	0.075	^*^
ΔPer Capita GDP	0.134	2.654	0.009	^**^
ECTt−1	−0.487	−4.21	0	^***^
**Stability and diagnostic tests**				
**Test**	χ^2^ **statistic**	***F*** **-statistic**	***P-*** **value**	
χ^2^_NORMAL	1.123	1.001	0.317	
χ^2^_SERIAL	2.345	2.123	0.204	
*χ^2^*_ARCH	1.789	1.654	0.276	
χ^2^_HETERO	2.21	2.045	0.248	
χ^2^_RESET	2.654	2.543	0.134	
CUSUM	Stable	–	–	
CUSUMSQ	Stable	–	–	
**Model fit and diagnostics**				
*R*^2^ = 0.812				
Adjusted *R*^2^ = 0.784				
Durbin-Watson = 2.067				

Dependent Variable: Average Expenditure Per Day of Domestic Tourists. ^***^, ^**^, and ^*^ represent level of significance at 1%, 5%, and 10%, respectively. Source: Author's estimations.

## 5 Robustness checks

To validate the long-run relationships identified through the Bootstrap ARDL (BARDL) approach, this study further employed Fully Modified Ordinary Least Squares (FMOLS) and Dynamic Ordinary Least Squares (DOLS) estimators. These methods correct for potential endogeneity and serial correlation, offering robust inference in cointegrated systems. The results are illustrated in Table 10.

**Table 10:** Robustness test results.

**Variable**	**FMOLS**				**DOLS**			
	**Coefficient**	**Std. Error**	***t*** **-Statistic**	***p-*** **Value**	**Coefficient**	**Std. Error**	***t-*** **Statistic**	***p*** **-Value**
Health Insurance	0.437	0.051	8.569	0.000	0.427	0.053	8.057	0.000
Health Expenditure	0.220	0.045	4.889	0.001	0.210	0.047	4.468	0.001
Avg. Exp. per Day	0.203	0.042	4.833	0.002	0.193	0.043	4.488	0.002
Income	0.392	0.048	8.167	0.000	0.382	0.050	7.640	0.000
Population	0.179	0.039	4.590	0.003	0.169	0.041	4.122	0.003
Per Capita GDP	0.261	0.044	5.932	0.001	0.251	0.046	5.457	0.001

The findings of Robustness Test Results using FMOLS and DOLS estimators are consistent with those in Table 6 (Bootstrapped ARDL Cointegration Analysis in the Long Run). All key variables remain statistically significant with positive coefficients across both methods, confirming the robustness of the long-run relationship between medical insurance and tourism turnover. This alignment reinforces the inference that improvements in health insurance coverage, public health expenditure, and economic conditions significantly contribute to the growth of domestic tourism in Vietnam.

## 6 Discussions and policy implications

This study provides robust empirical evidence on the long-run and short-run relationships between medical insurance and tourism development in Vietnam, using the Bootstrapped ARDL approach complemented by FMOLS and DOLS robustness checks. The findings confirm that health insurance coverage and public health expenditure significantly influence both macroeconomic tourism turnover and micro-level tourist spending. These results align with the broader literature that positions social protection not only as a safety net but also as a catalyst for discretionary consumption and sustainable tourism growth (4, 33).

### 6.1 Medical insurance as a driver of tourism consumption

The positive and statistically significant coefficients for health insurance across all models suggest that financial security provided by medical coverage encourages households to allocate resources toward leisure activities, including domestic travel. This supports the psychological assurance hypothesis, which posits that individuals with health protection are more confident in engaging in non-essential consumption (9, 10). In Vietnam, where health insurance coverage has reached near-universal levels (8), this dynamic is particularly relevant. The strong correlation between health insurance and tourism turnover (*r* = 0.850, *p* < 0.01) reinforces the idea that medical protection enhances household resilience and stimulates tourism demand.

These findings mirror those of Li et al. (3), who found that health insurance significantly boosts tourism consumption in urban China, especially among higher-income groups. Similarly, Dong and Li (4) demonstrated that insured households are more likely to increase tourism spending due to reduced financial uncertainty and improved psychological wellbeing. In Vietnam, this relationship is amplified by the country's rapid economic growth and expanding middle class, which collectively enhance the affordability and attractiveness of domestic travel.

### 6.2 Public health expenditure and destination appeal

State health expenditure also emerged as a significant determinant of tourism performance. Increased public investment in healthcare infrastructure not only improves population health but also enhances the perceived safety and accessibility of travel destinations. This is consistent with findings by Nguyen et al. (11), who emphasized that the integration of public and private health systems encourages preventive care and mobility, indirectly supporting tourism. In the post-COVID-19 context, health infrastructure has become a critical factor in destination choice, as tourists increasingly prioritize safety and medical accessibility (34).

Vietnam's expansion of healthcare services in both urban and rural areas has likely contributed to this trend. The positive impact of health expenditure on both tourism turnover and average daily spending suggests that public health investment plays a dual role—enhancing destination appeal and reinforcing consumer confidence.

### 6.3 Economic growth as a moderator

Per capita GDP was the most influential variable in the study, with consistently high coefficients and significance levels across all models. This finding aligns with global tourism literature, which identifies income levels as a primary determinant of travel behavior (12, 13). In Vietnam, GDP per capita has more than tripled over the past decade (35), enabling broader participation in domestic tourism and amplifying the effects of health insurance on consumption.

The moderating role of economic growth is particularly important. In periods of rising income, households are more likely to convert financial security into actual tourism spending. This suggests that the impact of medical insurance on tourism is not static but contingent on broader economic conditions. Policymakers should therefore consider the interplay between social protection and economic development when designing tourism strategies.

### 6.4 Short-run responsiveness and behavioral dynamics

The short-run results from the Bootstrapped ARDL model reveal that changes in health insurance, health expenditure, income, and GDP all positively influence tourism turnover and average daily spending. The error correction terms are negative and highly significant, confirming stable adjustments toward long-run equilibrium. These findings indicate that domestic tourism is responsive to short-term improvements in social protection and economic conditions.

Granger causality tests further validate the directional influence of medical insurance and GDP on tourism outcomes. Notably, average daily expenditure Granger-causes tourism turnover, but not vice versa, suggesting that shifts in individual spending behavior precede changes in aggregate tourism performance. This supports the conceptual framework proposed by Gu and Zhu (36), which emphasizes the role of household-level financial resilience in shaping broader economic activity.

### 6.5 Implications for policy and practice

The results of this study have several important implications for policymakers and stakeholders in Vietnam's tourism and health sectors.

Integrating Health and Tourism Policy: The strong link between medical insurance and tourism performance suggests that health policy should be considered a strategic component of tourism development. Investments in health coverage and infrastructure can yield broader economic benefits by stimulating domestic travel and leisure spending.

Promoting Inclusive Growth: While higher-income groups may benefit most from insurance-induced tourism spending, efforts should be made to extend these benefits to lower-income households. This could involve subsidized insurance schemes, targeted health investments in underserved regions, and community-based tourism initiatives that promote equitable access.

Enhancing Destination Safety and Appeal: Public health infrastructure plays a critical role in shaping tourist perceptions, especially in the wake of global health crises. Continued investment in healthcare services, emergency response systems, and health education can enhance Vietnam's reputation as a safe and accessible destination.

Leveraging Economic Growth: As GDP continues to rise, the government should capitalize on increased household purchasing power by promoting domestic tourism through marketing campaigns, infrastructure development, and digital platforms that facilitate travel planning and insurance access.

Supporting Tourism Resilience: Expanding unemployment insurance and other social protection mechanisms can help stabilize tourism demand during economic downturns. As (37) noted in the context of medical tourism in India, financial security is a key determinant of travel behavior. Strengthening these systems can enhance tourism resilience and support recovery in times of crisis.

## 7 Conclusion, contribution, limitation, and future research

This study provides robust empirical evidence that medical insurance and public health expenditure significantly influence domestic tourism development in Vietnam. Using the Bootstrapped ARDL approach, supported by FMOLS and DOLS robustness checks, the analysis confirms that health insurance coverage positively affects both tourism turnover and average daily expenditure by domestic tourists. These findings suggest that financial and psychological security—facilitated by medical protection—encourages discretionary spending on leisure activities. Furthermore, per capita GDP and public health investment amplify these effects, indicating that economic growth and infrastructure development are critical enablers of tourism responsiveness to social protection mechanisms.

The study contributes to the interdisciplinary literature by bridging health economics and tourism development, offering a novel perspective on how social protection policies can stimulate economic activity in non-essential sectors. Unlike previous research that focused on broader social insurance frameworks or high-income countries (4, 33), this study provides country-specific insights for Vietnam, where near-universal health insurance coverage has coincided with a surge in domestic tourism. Methodologically, the use of Bootstrapped ARDL alongside FMOLS and DOLS enhances the reliability of long-run inference, especially in small samples. The findings also offer policy-relevant insights, demonstrating that investments in health insurance and public health infrastructure can yield broader socio-economic benefits, including tourism stimulation and inclusive growth.

Despite its strengths, this study has several limitations. First, it relies on aggregate national-level data, which may obscure regional disparities and household-level variations in tourism behavior and insurance access. Second, the analysis focuses exclusively on domestic tourism, leaving the impact of medical insurance on international tourism unexplored. Third, while the study emphasizes medical insurance and public health expenditure, other dimensions of social protection—such as unemployment insurance or pension schemes—are not considered, though they may also influence tourism behavior. Fourth, the psychological and behavioral mechanisms through which insurance affects tourism are inferred rather than directly measured, which limits the depth of behavioral analysis. Fifth, although the study highlights psychological assurance and financial toxicity as potential mechanisms linking medical insurance to tourism behavior, these constructs are not empirically captured. Furthermore, omitted variables—such as cultural preferences, regional disparities, and local tourism infrastructure—may also shape both access to medical insurance and tourism behavior, potentially introducing unobserved heterogeneity.

Future research could address these limitations by employing micro-level survey data to examine how individual characteristics—such as age, education, health status, and income—interact with insurance coverage in shaping tourism decisions. Comparative analyses across provinces or between urban and rural areas could reveal heterogeneous effects of medical insurance. Moreover, future studies should investigate whether health insurance influences international tourism, particularly in the contexts of medical tourism and post-pandemic travel behavior. Exploring the role of digital health platforms and financial inclusion—such as mobile insurance services and digital finance (38)—could also yield valuable insights for innovation and policy design in Vietnam's evolving tourism landscape. In addition, survey-based or experimental methods could be employed to directly assess psychological factors such as perceived financial security, travel confidence, and risk aversion among insured and uninsured populations. Finally, incorporating regional-level data or qualitative approaches would enable a more comprehensive understanding of these dynamics.

## Data Availability

The original contributions presented in the study are included in the article/supplementary material, further inquiries can be directed to the corresponding author.
